# Do Upper and Lower Camptocormias Affect Gait and Postural Control in Patients with Parkinson's Disease? An Observational Cross-Sectional Study

**DOI:** 10.1155/2019/9026890

**Published:** 2019-07-24

**Authors:** Christian Geroin, Marialuisa Gandolfi, Isacco Maddalena, Nicola Smania, Michele Tinazzi

**Affiliations:** ^1^Neurology Unit, Movement Disorders Division, Department of Neurosciences, Biomedicine and Movement Sciences, University of Verona, Verona, Italy; ^2^Department of Neurosciences, Biomedicine and Movement Sciences, University of Verona, Verona, Italy; ^3^Neuromotor and Cognitive Rehabilitation Research Center (CRRNC), Department of Neurological and Movement Sciences, University of Verona, P.le L.A. Scuro 10, 37134 Verona, Italy

## Abstract

Gait impairments and camptocormia (CC) are common and debilitating in patients with Parkinson's disease (PD). Two types of CC affect patients with PD, but no studies investigated their relative contribution in worsening gait and postural control. Therefore, we investigated spatiotemporal gait parameters, gait variability, and asymmetry and postural control in PD patients (Hoehn & Yahr ≤4) with upper CC and lower CC and patients without CC. This observational cross-sectional study involving patients with PD and upper CC (*n*=16) and lower CC (*n*=14) and without CC (*n*=16). The primary outcome measure was gait speed assessed by the GAITRite System. The secondary outcome measures were other spatiotemporal parameters, gait variability, and asymmetry. Postural control and balance were assessed with posturography and the Mini-BESTest. Patients with lower CC showed a higher H&Y stage (*p*=0.003), a worse PDQ8 (*p*=0.042), and a lower Mini-BESTest score (*p*=0.006) than patients with PD without CC. Patients with lower CC showed a reduced gait speed (*p*=0.012), stride length, and velocity than patients with PD without CC. Upper CC patients showed a higher stride length than lower CC ones (*p*=0.007). In the eyes open and closed condition, patients with lower CC showed a higher (worse) velocity of CoP displacement in mediolateral direction and length of CoP than patients with PD without CC. No significant between-group differences were measured in gait variability and asymmetry. In conclusion, lower CC was associated with more severe gait and postural control impairment than patients with upper CC and without CC. Categorizing CC based on the bending fulcrum is compulsory to identify patients with the worst performance and to implement specific rehabilitation programs.

## 1. Introduction

Gait impairments and camptocormia (CC) are common and debilitating in patients with Parkinson's disease (PD) [1–6]. They impose substantial disability on these patients, increasing the risk of falling, and related injuries, and reducing the quality of life [1–6]. According to a recent conceptual model, gait disturbances can be characterized using a principal component analysis in five independent domains: pace, rhythm, variability, asymmetry, and postural control domains [7]. In PD, the three principal gait impairments (gait slowness, increased variability, and postural control deficits) fall into these domains [5].

In the current literature, the influence of postural abnormalities on gait disturbances has been rarely explored. On the one hand, it depends on the fact that a consensus of diagnostic criteria on postural abnormalities in PD has been only recently reached. Pisa syndrome was defined as at least 10° lateral flexion of the trunk, which typically resolves by passive mobilisation or supine positioning [3]. Antecollis relates to forward flexion of the neck (minimum 45°) [3]. Finally, camptocormia (CC) has been recently fully characterised as a sagittal plane deformity originating either in the thoracic or lumbar spine appearing during standing or walking and resolving in the supine position [2].

On the other hand, CC has been incorrectly considered as a single entity. Nowadays, a consensus has been reached in differentiating forward trunk flexion in lower and upper camptocormias. The former refers to “an involuntary flexion of the spine of at least 30° at the lumbar fulcrum (L1-Sacrum).” The latter refers to “an involuntary flexion of the spine of at least 45° at the thoracic fulcrum (C7 to T12-L1)” [2]. This additional classification allows the clinician to define deformities in the sagittal plane better and then to investigate whether the different types of CC would impose specific disability in patients with PD.

So far, only two studies have explored the influence of postural abnormalities in gait dynamics and postural control [8, 9]. Geroin et al. reported for the first time that patients with Pisa syndrome (PS) showed higher (worse) postural instability than age-matched patients with PD but without PS and healthy controls (irrespective of side and severity). Patients with PD and PS reported a significantly higher velocity of the Center of Pressure (CoP) displacement in the mediolateral and anteroposterior directions than the other two groups, with the worst performance in the eyes, closed condition. No significant differences were reported on spatiotemporal gait parameters among groups [8]. In a recent observational cross-sectional study, Tramonti et al. investigated gait dynamics using 3D Gait analysis and clinical scales in patients with PD and PS, with CC, and without postural deformities. Gait speed, stride, and step length decreased in patients without postural abnormalities and PS and CC groups compared to healthy subjects. Functional abilities and disease severity were worse in the PS and CC patients than patients without postural abnormalities. Kinematic data revealed a marked reduction in the lower-extremity range of motion (ROM) in the patients with PS. However, the CC group showed a more noticeable reduction in hip and knee joints range of motion suggesting an increased hip flexion pattern during gait [9]. The main study limitation is the lack of distinction between upper and lower CC. The diagnosis of CC should take into account both the bending angle and fulcrum to be correctly categorised and differentiated from a generically stooped posture [2].

To our knowledge, no studies to date have explored the relative contribution to gait impairment and postural control of the upper and lower CC in patients with PD. Moreover, gait variability and asymmetry have not been previously investigated in these populations. The primary aim of this study was to investigate gait speed differences in patients with PD with upper and lower CC and patients with PD without CC.

The secondary aim was to investigate changes in the other spatiotemporal gait parameters according to the conceptual models of gait [7] between patients with PD with upper and lower CC and patients with PD without CC. We hypothesized that patients with lower CC would be more affected than other groups in both gait and postural control due to biomechanical constraints to the lumbar/sacral region.

## 2. Materials and Methods

### 2.1. Study Design and Setting

An observational cross-sectional study involving patients with PD with upper CC and lower CC and without CC (PD) was conducted. Patients were recruited from the outpatient's clinic of the Movement Disorders Division and the UOC Neurorehabilitation Unit of the University Hospital (AOUI Verona, Italy) from March 2018 to October 2018.

### 2.2. Participants

Forty-six patients with PD (mean age 70.9 ± 6.6) were divided into three groups: patients with upper CC (*n*=16), lower CC (*n*=14), and without CC (*n*=14). The severity of forward trunk flexion was evaluated using a software-based measurement of the undressed (with underwear) body patients' pictures. The lateral view pictures of the patients were taken with the camera lens at approximately waist level. The measurements were performed by an experienced rater using a freeware program Kinovea® [10].

Patients were diagnosed with CC when presenting an “involuntary flexion of the spine appearing during standing or walking and resolving in the supine position of at least 30° at the lumbar fulcrum (L1-sacrum and hip flexion, i.e., lower CC) or at least 45° at the thoracic fulcrum (C7 to T12-L1, i.e., upper CC)” [2].

At the enrolment, all patients underwent a neurological screening and physical examination. Inclusion criteria were age ≥18 years old; clinical diagnosis of PD according to MDS clinical diagnostic criteria [11]; Hoehn & Yahr (H&Y) stage ≤4 in the “ON” medication phase and on their usual antiparkinsonian treatment. Exclusion criteria were severe dyskinesia or “on-off” fluctuations; PD medication modification in the 3 months preceding the enrolment; the presence of PS [3]; a history of major spinal surgery or muscle and/or skeletal spine diseases (namely, vertebral fractures, spondylodiscitis, and inflammatory myopathy); need for assistive devices to rise from a chair or bed; other neurological (i.e., vertigo and vestibular disorders), orthopedic, or cardiovascular comorbidities that could interfere with gait; and ability to walk for at least 10 meters without the use of device. Patients gave their written, informed consent after being informed about the experimental nature of the study. The authorization has been obtained for disclosure (consent-to-disclose) of any recognizable persons in photographs. The study was carried out following the Helsinki Declaration, approved by the local Ethics Committee (prog. no. 2399).

### 2.3. Testing Procedures

Demographic and clinical variables were collected by an MDS specialist and included age, gender, Unified Parkinson's Disease Rating Scale total score and Part III (UPDRS III), H&Y stage, PD phenotype (rigid-akinetic, tremor-dominant, or mixed type) [12], Montreal Cognitive Assessment (MOCA) Score [13]; Parkinson's Disease Questionnaire-8 Score (PDQ8) [14], the number of falls in the previous month [15], the Mini-BESTest [16], and the Numeric Rating Scale (NRS) to quantify back pain.

All patients underwent instrumental gait assessment using the GAITRite walkway system (CIR Systems Inc, Havertown, PA) 7.92 m in length and sampling at a frequency of 120 Hz. The patients walked at a self-selected comfortable speed without walking aids. The data from the three trials were collected, and their average was calculated. Gait parameters were selected following a model developed in older adults and validated in PD composed of five domains [7, 17]: (1) pace domain: gait speed (cm/s), stride, and step length (cm), width of base of support (cm), and stride velocity (cm/sec); (2) rhythm domain: cadence (step/min), step time (sec), swing time (sec), stance time (sec), single support time (sec), and double support time (sec); (3) phases: swing %, stance %, single %, and double support % of gait cycle; (4) asymmetry domain: step length and stance time calculated as the absolute difference between left and right step means; (5) variability measures were quantified using the coefficient of variation, e.g., stride length variability = 100 × (SD of stride length/average stride length) [18, 19]. The coefficient of variability for the stride length, base of the support, double support time, and stride velocity was computed as related to falling in older adults [20].

**Figure 1:**
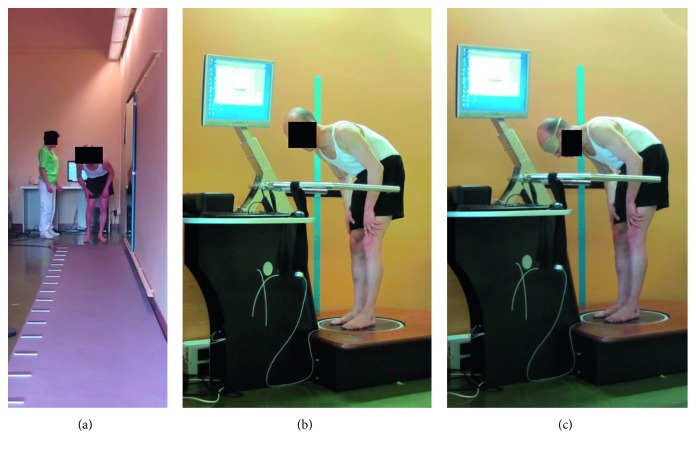
A patient with lower CC during the gait (a) and posturographic assessment with eyes open (b) and eyes closed condition (c).

Posturography was performed in the standing position on an electronic monoaxial platform (Technobody©). The feet position on the platform was standardized using a V-shaped frame for all patients. The distance between the two malleoli was 3 cm, and the medial borders of the feet were extra rotated 12° with respect to the anteroposterior axis. The patients were evaluated while standing upright without the use of upper limb support in the eyes open (EO) and the eyes closed (EC) condition, each lasting 30 s [8]. The following outcomes were recorded: the velocity of the CoP displacement in the anteroposterior and mediolateral direction (mm/sec), length of CoP trajectory (mm), and sway area (mm^2^) (Figure 1).

The primary outcome measure was gait speed while secondary outcome measures were other spatiotemporal parameters, gait variability and asymmetry, and stabilometric outcomes.

### 2.4. Statistical Analysis

Descriptive statistics included calculation of frequency tables, means, and standard deviation. Absolute and relative frequencies were calculated for categorical data and tested by Fisher's Exact test after checking the minimum acceptable number of expected frequencies (<5). Variables were tested for normality with the Shapiro–Wilk test. When the continuous variables were normally distributed, the comparisons across groups (PD vs upper CC vs lower CC) were performed with parametric tests. The equality of variances (homogeneity) was checked using Levene's test. If variances were heterogeneous, we used Welch's ANOVA test, otherwise the one-way ANOVA. The post hoc comparisons were performed with the Tukey test. When the continuous variables were not normally distributed, the comparisons across groups (PD vs upper CC vs lower CC) were performed with nonparametric Kruskal–Wallis H test. The post hoc comparisons were performed with the Mann–Whitney *U* test.

Further, Pearson's or Spearman's coefficient was used to analyze the correlations between spatiotemporal gait parameters (gait speed and stride length), posturographic parameters (eyes open/close velocity of mediolateral CoP displacements and length of CoP), and H&Y stage in the three groups. All tests were bilateral at *p* < 0.05. Statistical analysis was carried out using the SPSS for Mac statistical package, version 20.0.

## 3. Results

Patients recruited were receiving chronic therapy with a dopaminergic drug and showed good motor compensation in appendicular function. None had psychiatric disturbances. Patients with upper CC had a forward trunk flexion of 47.64 ± 2.66°, and 8 showed a back pain with NRS of 3.2 ± 1.7. Patients with lower CC had a forward trunk flexion of 48.24 ± 13.85°, and 9 showed a back pain with NRS of 4.7 ± 2.2. Patients without CC had a forward trunk flexion of 19.12 ± 20.25°, and 8 showed a back pain with NRS of 3.1 ± 1.4.

We found a main effect for the H&Y (*F* = 5.04; *df* = 2, *p*=0.011), PDQ8 (*p*=0.043), and the Mini-BESTest (*F* = 5.55; *df* = 2, *p*=0.007) (Table 1). Post hoc analysis revealed a significant difference between PD and patients with lower CC in the H&Y stage (*p*=0.003), PDQ8 (*p*=0.042), and Mini-BESTest (*p*=0.006).

### 3.1. Primary Outcome Measures

A significant main effect in the gait speed (*F* = 5.37; *df* = 2, *p*=0.011) was measured. Post hoc analysis revealed that patients with lower CC had a significantly reduced gait speed than patients with PD (*p*=0.012).

### 3.2. Secondary Outcome Measures

A significant main effect in the stride length (*p* < 0.001), step length (*p* < 0.001), and stride velocity (*F* = 5.39; *df* = 2, *p*=0.011) was reported. Post hoc analysis revealed a significant difference in stride length between PD and patients with lower CC (*p* < 0.001) and between patients with lower CC and upper CC (*p*=0.007). In step length, a significant difference between PD and patients with lower CC (*p* < 0.001) and between patients with lower CC and upper CC (*p*=0.008) was measured. Patients with lower CC showed a significant shorter stride and step length than patients with PD and upper CC. In stride velocity, post hoc analysis revealed a significant difference between PD and patients with lower CC (*p*=0.012). Post hoc analysis revealed that patients with lower CC had a significant slower stride velocity than patients with PD. No statistically significant results were reported in the other spatiotemporal gait parameters.

In the eyes open condition, a significant main effect in the velocity of CoP in the mediolateral direction (*p*=0.004) and length of CoP (*p*=0.019) was reported. Post hoc analysis revealed a significant difference between PD and patients with lower CC in the velocity of CoP (*p*=0.003) and the length of CoP (*p*=0.014).

Similarly, a significant main effect in the velocity of CoP in mediolateral direction (*p*=0.011) and length of CoP (*p*=0.015) was measured in the eyes closed condition. Post hoc analysis revealed a significant difference between PD and patients with lower CC in the velocity of CoP (*p*=0.009) and the length of CoP (*p*=0.014).

In eyes open and closed condition, patients with lower CC revealed a higher velocity of CoP in mediolateral direction and length of CoP than patients with PD. We did not find any other statistically significant results.

No significant correlation coefficients were found between spatiotemporal gait parameters (gait speed and stride length), posturographic parameters (eyes open/closed velocity of mediolateral CoP displacements and length of CoP), and H&Y stage in the three groups.

## 4. Discussion

The main finding of this study is that the patient with lower CC exhibited the highest degree of gait and postural control impairment. Our data extend previous data on the influence of CC on functional performance during walking and, for the first time in the literature, showed that the two types of CC may affect (or not) gait and postural control [2, 9].

According to the literature [2, 3], the presence of CC was associated with higher neurological severity, worse balance performance, and quality of life than patients without CC, as reported in Table 1. However, only patients with lower CC reported scores significantly worse than patients without CC. Gait analysis and postural assessment showed that lower CC was associated with a significant reduction in performance in the pace domain (except for the width of the base of support). Besides, a significant increase in the velocity of the CoP displacement in mediolateral direction and length of CoP in both eyes open and closed conditions was reported. This finding was significantly different between upper and lower CC strengthening, the hypothesis that lower CC affects gait more than the upper type. Thus, the forward trunk flexion by lower fulcra may be the most disabling postural abnormalities in patients with PD.

CC is not a levodopa-responsive abnormality that can be (before being more fixed) fully reversible in the supine position and using manoeuvres like “sensory tricks” (i.e., the patients to stand up straight or against a vertical reference) [4]. The existing evidence suggests that CC may have multifactorial pathophysiology involving central and peripheral hypotheses [3, 4]. The former, supported by animal and clinical studies, takes into account an asymmetric functioning of basal ganglia output leading to asymmetric control of trunk muscles tone (dystonia) along with an altered internal model of postural perception [4]. The latter considers CC as a consequence of paraspinal myopathy due to the pathophysiology of PD. However, this possibility needs to be further investigated [4]. Distinct muscles patterns might be involved in the bimodal distribution of forward trunk flexion. In the upper CC, a bilateral overactivity of abdominal external and internal oblique along with rectus abdominis muscles has been described [4, 21–23].

In contrast, in the lower subtype, combined activation of rectus abdominis and iliopsoas muscles has been reported [4]. Our finding suggested two mutually nonexclusive hypotheses. From a biomechanical perspective, the lower CC may compromise the iliopsoas function. As reported by the physiological literature, the iliopsoas muscle flexes the femur in the standing position and acts as a stabilizer of the femoral head in the hip acetabulum in the first 15° of movements. Finally, it maintains the director action from 15° to 45° degrees and acts as an effective flexor of the femur from 45 to 60° [24]. The reduced stride length and gait speed found in patients with lower CC might be explained by the pathological flexion of the trunk during gait limiting the hip extension. The reduction of hip extension, indeed, is a primary factor in the reduction of the ROM at the hip, step length, and gait speed [5, 9]. Moreover, the excessive flexor muscle activity at the knee and ankle further reduced lower limb joint torques during walking [6].

From a neurological perspective, gait slowness may be the result of more severe hypokinesia (reduced step size), bradykinesia (increased step duration), and axial rigidity. It would explain why patients with lower CC displayed a severe neurological severity, as measured by the H&Y stage.

Walking can be understood as a repeated sequence of the centre of mass displacements to maintain lateral and forward stability [6]. A decrease of gait speed is a self-imposed compensatory strategy to maintain balance during walking in PD. The low gait speed observed in patients with lower CC can be related to a worsening of balance control, as measured by the mediolateral CoP displacement. The abnormal flexed posture observed in lower CC pushes the CoP forward the base of support at the limits of stability. The literature emphasised that the lateral control of balance is impaired in patients with PD showing elevated lateral trunk sway during stance and walking [5] and it is associated with falls [6]. Patients with lower CC might be less prone to sway in the anteroposterior direction than in the mediolateral direction because of the hyperflexed posture limiting the hip range of motion in the anteroposterior direction. As a consequence, the patient with lower CC reported a higher number of falls than the other two groups, albeit not significant.

The three groups were comparable in gait variability and asymmetry, suggesting that these domains might be independent of the CC and related to the disease severity itself [6]. According to the literature, our results suggest that gait variability is independent of gait speed, cadence, and stride length [25]. An increase in gait variability in PD is expected in comparison with healthy controls presumably related to basal ganglia dysfunction and not to CC [25, 26]. Gait speed and stride length parameters showed in our PD patients were similar to findings reported in older adults [27]. It suggests that the stage of disease and phenotype have a primary role in impairing gait and balance in PD.

The main study limitation is the lack of 3D gait analysis to assess trunk and lower limbs during gait quantitatively. Larger sample size may strengthen the statistics of the study and display significant differences among groups not found in our preliminary report.

## 5. Conclusions

Lower CC was associated with more severe gait and postural control impairment than upper CC and without CC. Categorizing CC based on the bending fulcrum is compulsory to identify patients with the worst outcome and to implement specific rehabilitation programs. Future rehabilitation studies are needed to assess the rehabilitation effects on the severity of the forward trunk flexion and postural control in patients with lower camptocormia (Tables 2 and 3).

## Figures and Tables

**Table 1:** Demographic and clinical characteristics of the patients.

	Total Group	CC Upper	CC Lower	PD	*p* Value
Patients, no.	46	16	14	16	
Age, mean (SD), yrs	70.9 (6.6)	71.6 (4.36)	70.3 (8.21)	70.7 (7.3)	0.787^c^
Gender, M/F	31/15	12/4	7/7	12/4	0.283
UPDRS total score	53.1 (23.9)	53 (30.2)	59.6 (18.4)	47.5 (21.1)	0.250^c^
UPDRS III score	29.7 (14.3)	29.2 (17.6)	33.9 (11.9)	26.4 (12.2)	0.283^c^
H&Y stage	2.2 (0.8)	2.2 (0.9)	2.6 (0.6)	1.8 (0.6)	**0.011** ^*∗*a^
Dominant phenotype, *n* (%)					0.131^c^
Tremor type	11 (24)	5 (31.2)	1 (7.2)	5 (31.2)	—
Bradykinetic/rigid type	29 (63)	7 (43.8)	12 (85.7)	10 (62.5)	—
Mixed type	6 (13)	4 (25)	1 (7.1)	1 (6.3)	—
MoCA	24.3 (3.3)	23.7 (3.9)	24.1 (3.1)	25.2 (3.1)	0.545^c^
PDQ8	20.1 (13.3)	18.2 (11.8)	25.9 (13.1)	16.8 (14)	**0.043** ^*∗*a^ ^c^
Falls	1.1 (1.9)	1.2 (2.5)	1.6 (1.8)	0.5 (0.9)	0.175^c^
Mini-BESTest	19.6 (5.6)	19.1 (6.5)	16.6 (4.7)	22.7 (3.6)	**0.007** ^*∗*a^

CC denotes patients with Parkinson's disease and camptocormia according to consensus-based diagnostic criteria (Fasano2018); PD, patients with Parkinson's disease (without CC); SD, standard deviation; M, Male; F, Female; yrs, years; UPDRS, Unified Parkinson's Disease Rating Scale; UPDRS III, subitem of UPDRS scale part III; H&Y, Hoehn and Yahr stage; MoCA, Montreal Cognitive Assessment; PDQ8, Parkinson' s Disease Questionnaire-8; Falls, number of falls in the previous month; ^a^Welch's ANOVA test; ^b^Fisher's exact test; ^c^Kruskal–Wallis *H* test; *p* significant if < .05; values with ^*∗*^ and in bold are considered statistically significant.

**Table 2:** Multiple pairwise comparisons between the three groups for each outcome measure.

Spatiotemporal gait parameters	CC Upper	CC Lower	PD	*p* Value main effect
*Pace domain*				
Gait speed (cm/s)	96.27 (16.62)	79.05 (20.74)	108.55 (30.90)	**0.011** ^*∗*^
Stride length (cm)	106.27 (15.36)	83.61 (4.74)	115.26 (21.93)	**<0.001** ^*∗*^
Step length (cm)	53 (7.69)	41.67 (8.81)	57.42 (10.95)	**<0.001** ^*∗*^
Width of base support (cm)	8.77 (3.04)	9.71 (3.91)	8.95 (3.07)	0.725
Stride velocity (cm/s)	97.05 (16.55)	79.81 (20.91)	109.66 (31.05)	**0.011** ^*∗*^

*Rhythm domain*				
Cadence (step/min)	109.04 (10.89)	113.10 (15)	111.94 (13.23)	0.679
Step time (sec)	0.55 (0.06)	0.54 (0.07)	0.54 (0.06)	0.723
Swing time (sec)	0.42 (0.04)	0.38 (0.04)	0.40 (0.03)	0.149
Stance time (sec)	0.69 (0.07)	0.68 (0.11)	0.68 (0.09)	0.924
Single support time (sec)	0.42 (0.04)	0.38 (0.04)	0.40 (0.03)	0.149
Double support time (sec)	0.28 (0.04)	0.30 (0.10)	0.28 (0.08)	0.966

*Phases*				
Swing % of gait cycle (%)	37.48 (1.43)	36.26 (3.22)	37.31 (2.62)	0.673
Stance % of gait cycle (%)	62.52 (1.44)	63.73 (3.22)	62.70 (2.62)	0.687
Single support % of cycle	37.49 (1.41)	36.25 (3.23)	37.32 (2.61)	0.643
Double support % of cycle	25.07 (2.87)	27.35 (6.51)	25.36 (5.12)	0.704

*Asymmetry*				
Step length difference (cm)	4.12 (2.65)	2.97 (2.11)	2.36 (1.51)	0.072
*Stance time difference (sec)*	0.01 (0.03)	0	0	0.365

*Coefficient of variability*				
Stride length, CV	5.18 (1.96)	5.83 (2.62)	4.77 (2.11)	0.479
HH base support, CV	22.88 (12.79)	22.97 (13.33)	24.06 (11.11)	0.957
Double support time, CV	14.81 (11.08)	13.24 (7.06)	13.65 (7.92)	0.995
Stride velocity, CV	7.74 (2.89)	9.24 (4.29)	7.78 (2.95)	0.674

CC denotes patients with Parkinson's disease and camptocormia according to consensus-based diagnostic criteria [2]; PD, patients with Parkinson's disease (without CC); *p* significant if <0.05; values with ^*∗*^ and in bold are considered statistically significant.

**Table 3:** Multiple pairwise comparisons between the three groups for each posturography measure.

Posturography	CC Upper	CC Lower	PD	*p* Value main effect
*Variables eyes open*				
VEL_MED_AP (mm/sec)	4.25 (2.08)	5.78 (3.55)	3.56 (1.31)	0.086
VEL_MED_ML (mm/sec)	3.25 (1.34)	4.57 (2.03)	2.56 (1.09)	**0.004** ^*∗*^
Length CoP (mm)	149.12 (62.92)	206.50 (106.65)	121.18 (43.90)	**0.019** ^*∗*^
Sway area (mm^2^)	93.62 (108.71)	125.14 (110.33)	79.56 (61.72)	0.518

*Variables eyes closed*				
VEL_MED_AP (mm/sec)	6.18 (2.76)	7.78 (5.21)	4.37 (1.63)	0.050
VEL_MED_ML (mm/sec)	4.56 (1.78)	6.28 (3.45)	3.37 (1.74)	**0.011** ^*∗*^
Length CoP (mm)	215.50 (86.26)	282.21 (163.26)	157.25 (59.65)	**0.015** ^*∗*^
Sway area (mm^2^)	168.44 (171.36)	181.86 (122.67)	113 (125.57)	0.069

CC denotes patients with Parkinson's disease and camptocormia according to consensus-based diagnostic criteria [2]; PD, patients with Parkinson's disease (without CC); CoP, centre of pressure; VEL_MED_AP, velocity of anteroposterior CoP displacement; VEL_MED_ML, velocity of mediolateral CoP displacement; *p* value, Kruskal–Wallis test; *P* significant if <0.05; values with ^*∗*^ and in bold are considered statistically significant.

## Data Availability

The data used to support the findings of this study are available from the corresponding author upon request.
